# Differential olfactory bulb methylation and hydroxymethylation are linked to odor location memory bias in injured mice

**DOI:** 10.1177/1744806919873475

**Published:** 2019-08-27

**Authors:** Maral Tajerian, Sebastian G Alvarado, J David Clark

**Affiliations:** 1 Department of Biology, Queens College, City University of New York, Queens, NY, USA; 2 Veterans Affairs Palo Alto Health Care System, Palo Alto, CA, USA; 3 Department of Anesthesiology, Stanford University School of Medicine, Stanford, CA, USA; 4 Palo Alto Veterans Institute for Research, Palo Alto, CA, USA

**Keywords:** Chronic pain, DNA methylation, DNA hydroxymethylation, olfactory bulb, preclinical models, epigenetics, memory

## Abstract

Chronic pain is often linked to comorbidities such as anxiety and cognitive dysfunction, alterations that are reflected in brain plasticity in regions such as the prefrontal cortex and the limbic area. Despite the growing interest in pain-related cognitive deficits, little is known about the relationship between the emotional valence of the stimulus and the salience of its memory following painful injuries. We used the tibia fracture model of chronic pain in mice to determine whether pleasant and unpleasant odor location memories differ in their salience seven weeks following the onset of the painful injury. Our results indicate that injured mice show a bias toward recalling unpleasant memories, thereby propagating the vicious cycle of chronic pain and negative affect. Next, we linked these behavioral differences to mechanisms of molecular plasticity by measuring the levels of global methylation and hydroxymethylation in the olfactory bulb. Compared to controls, global methylation levels were shown to be increased, while hydroxymethylation levels were decreased in the olfactory bulb of injured mice, indicative of overall changes in DNA regulation machinery and the subsequent alterations in sensory systems.

In clinical populations, chronic pain is often accompanied by alterations in affect, cognition, and overall sensory processing.^1,2^ Similarly, memory deficits have been reported in animal models of pain^3,4^ and linked to cellular^5^ and extracellular^6^ mechanisms of brain plasticity. However, the question of whether chronic pain consistently results in overall cognitive deficits remains unresolved since the reported cognitive decline in preclinical models is highly dependent on the behavioral assay used.^5^

One reason for this apparent inconsistency in findings could be due to the valence of memories studied. When interpreted within the scope of content specificity theory of information processing, it is likely that the unpleasant versus pleasant stimuli are consolidated via different processes and could differ in the manner by which they are recalled or reconstructed. For instance, compared to pain-free controls and nondepressed pain patients, depressed pain patients were shown to display a bias toward self-referential negative pain words.^7^ In a more recent clinical study of chronic pain patients, memory bias toward negatively emotionally valenced memories was reported.^8^ We therefore set out to test the hypothesis that the negative valence of the initial exposure is linked to increased salience of the memory in chronic pain versus control mice.

Most rodent studies of negatively valenced memories utilize noxious stimuli such as electric shock, an approach that is not well suited for a study where painful limb injury with potential peripheral nerve damage is the experimental variable. We therefore used an unpleasant odor and compared it to a pleasant odor in our behavioral paradigms. Our results support the hypothesis that injured mice show a partiality to recall unpleasant memories, thereby contributing to the vicious cycle of negative affect and biased memories.^9^ Furthermore, our analysis of the olfactory bulb (OB) shows lasting epigenetic alterations in terms of DNA methylation and hydroxymethylation which parallel these behavioral findings.

Male C57BL/6J mice ages 12–14 weeks were purchased from a commercial supplier (Jackson Labs, Sacramento, CA, USA) and were allowed to habituate to the animal facility for a minimum of 10 days prior to the experiments. Mice were housed in groups of four on a 12-h light/dark cycle and an ambient temperature of 22 ± 3°C, with food and water available ad libitum. All animal procedures and experimental designs were approved by the *Veterans Affairs Palo Alto Health Care System Institutional Animal Care and Use Committee* (Palo Alto, CA, USA) and followed the “animal subjects” guidelines of the *International Association for the Study of Pain.*

A total of three cohorts of mice was used. Following the random allocation to the control or the fracture/cast group, mice were anesthetized with 1.5% isoflurane and underwent a distal tibial fracture in the right leg. Briefly, a hemostat was used to make a closed fracture of the right tibia just distal to the middle of the tibia and the hindlimb was wrapped in casting tape (cat. # 82001, Scotchcast™ Plus, 3 M, UK), as previously described.^10^ After the procedure, the mice were given subcutaneous buprenorphine (0.05 mg/kg) and enrofloxacin (5 mg/kg) for the next two days, as well as normal saline (1.5 ml once) for postoperative analgesia, prevention of infection, and prevention of dehydration. Mice were inspected daily to ensure the cast was positioned properly through the three-week period of cast immobilization. Mice were provided with chow pellets postoperatively ad libitum; dietary gels were also made available on the cage floor for mice having undergone surgery. Casts were removed three weeks after surgery under brief isoflurane anesthesia.

Behavioral testing was carried out in the first cohort of mice by a blinded observer seven weeks following injury (Figure 1(a)). Mechanical sensitivity was measured using the von Frey filament method where calibrated monofilaments (Stoelting Co., USA) were applied to the plantar surface of the hindpaw, and the 50% threshold to withdraw (grams) was calculated as previously described.^11^ Seven weeks following fracture, injured mice displayed significantly lower thresholds compared to control mice (two-tailed Student’s *t*-test, significance was set at P value <0.05 (Prism 5; GraphPad Software, USA), Figure 1(b)). These data are in agreement with the multiple studies published in this model of chronic pain.^5,6,12–18^

**Figure 1.**
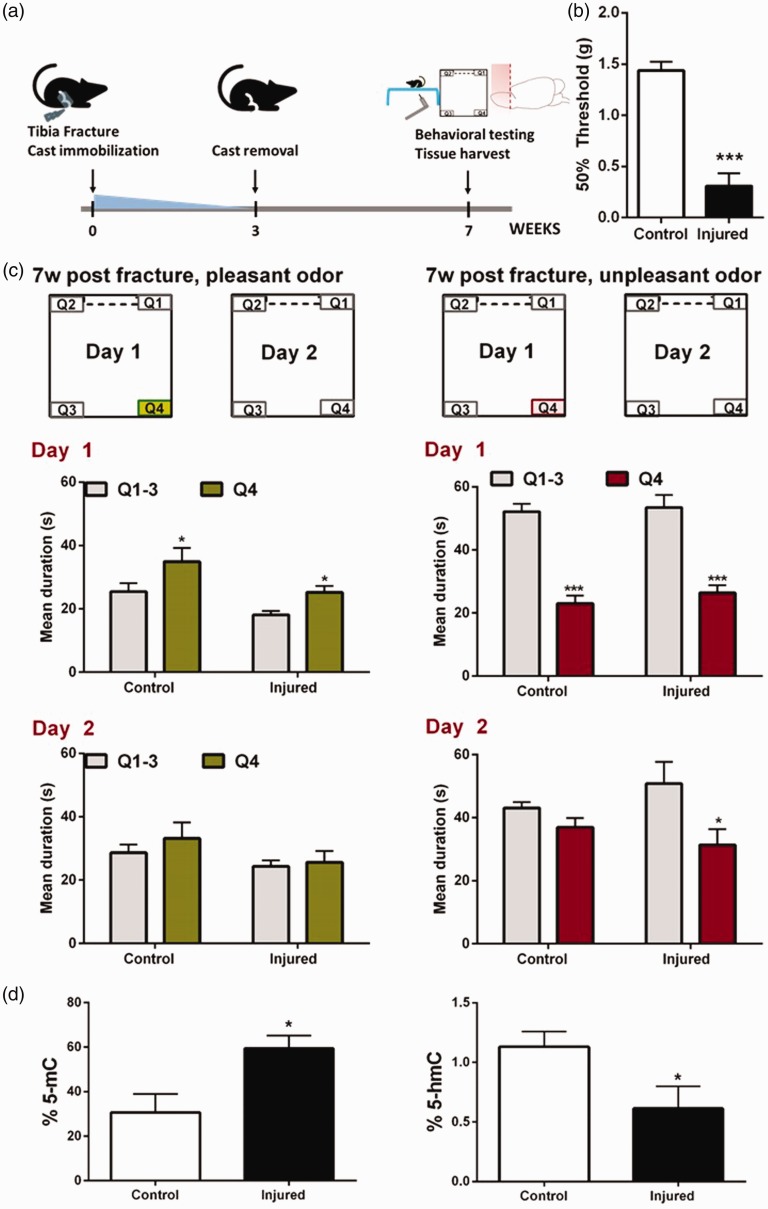
(a) Experimental timeline. (b) Mechanical sensitivity: compared to control animals, injured mice display decreased mechanical thresholds on the ipsilateral hindpaw seven weeks following fracture. Two-tailed Student’s *t*-test, n = 6 mice/group. Error bars indicate SEM. (c) Odor location memory. *Pleasant odor*: Day 1: Both injured and control animals spend more time exploring the object containing Linalool. Day 2: both injured and control animals spend equal amounts of time exploring each of the objects. *Unpleasant odor*: Day 1: both injured and control animals spend less time exploring the object containing 2-methylbutyric acid. Day 2: while control animals spend equal amounts of time exploring each of the objects, injured mice continue to avoid the quadrant where the object containing the 2-methylbutyric acid was previously located. Two-way ANOVA followed by Holm–Sidak post hoc test for multiple comparisons, n = 9–12 mice/group. Error bars indicate SEM. (d) Olfactory bulb methylation and hydroxymethylation. Injured mice display increased levels of global methylation and decreased levels of hydroxymethylation in the olfactory bulb seven weeks after fracture. Two-tailed Student’s *t*-test, n = 4–6 mice/group. Error bars indicate SEM. OLM: odor location memory; Q: quadrant; mC: methylcytosine; HmC: hydroxymethylcytosine.

For odor memory testing, we utilized an arena that was a square box 45 cm × 45 cm × 45 cm (L × W × H) made of white polyvinyl chloride. Four plastic objects were placed at each of the arena corners. Mice were placed into the arena from the middle of the south wall, with the north wall of the arena having a large visual cue (28 cm × 21.5 cm [W × H] sheet with alternating black and white columns (column width = 1.5 cm)). Mice underwent one 10-min experience trial and a recall trial 24 h later. During the experience trial, one of the four plastic objects contained either a pleasant or unpleasant odorant molecule (gauze pad with 200 µl of either linalool (Sigma Aldrich) or 2-methylbutyric acid (Sigma Aldrich), respectively). The two odorants were chosen based on a set of preliminary studies examining the valence of 10 odorant compounds. No odorant compounds were present during the recall trial. Two identical sets of arenas were used for the two trials to ensure the absence of odors during the recall trial. A different cohort of mice (cohorts 2 and 3) was used for each of the two odorants.

Analysis of repeated parametric measures was accomplished using a two-way analysis of variance (ANOVA) followed by Holm–Sidak post hoc test for multiple comparisons (significance was set at P value <0.05 (Prism 5; GraphPad Software)). Our data show valence-dependent alterations in odor location memory in injured mice. When testing for the pleasant OLM, injured and control mice did not differ in their total exploration time of all objects (Q1–Q4) on either day of testing. On day 1, both injured and control mice spent more time exploring the object containing the pleasant odor (Q4). On day 2, in the absence of the pleasant odor in Q4, injured or control mice showed no preference toward exploring Q4 (Figure 1(c)). Similarly, when testing for the unpleasant OLM, injured and control mice did not differ in their total exploration time of all objects (Q1–Q4) on either day of testing. On day 1, both injured and control mice spent less time exploring the object containing the unpleasant odor (Q4). In contrast to outcomes from the pleasant OLM study, on day 2, in the absence of the unpleasant odor in Q4, only injured mice showed an aversion toward exploring Q4 (Figure 1(c)). These findings agree with previous data where neuropathic mice were unable to extinguish to contextual fears,^19^ suggesting that unpleasant memories are slow to decay in a pain state.

To link these behavioral differences to mechanisms of molecular plasticity, we measured global methylation and hydroxymethylation levels in the OB in the first cohort of mice (which were used for mechanical sensitivity testing). Within a week after behavioral testing, mice were sacrificed under isoflurane anesthesia and the OB was quickly harvested and stored at −80°C until use. Following DNA isolation (cat. #156892, Abcam, USA), DNA methylation (cat. #117129, Abcam), and hydroxymethylation (cat. #117131, Abcam) were quantified per the manufacturer’s instructions. Compared to controls, global methylation levels were shown to be increased, while hydroxymethylation levels were decreased in the OB of injured mice, seven weeks after fracture (two-tailed Student’s *t*-test, significance was set at P value <0.05 (Prism 5; GraphPad Software), Figure 1(d)).

Recent findings inform us of a significant overlap between the two sensory modalities of olfaction and nociception.^20^ Case studies link chronic pain and sodium channel mutations to increased olfactory sensitivity,^21^ and maternal milk odor was shown to be analgesic in infants.^22^ Furthermore, it has been shown that exposure to certain odorants such as linalool, the pleasant odorant in our study, have OB-dependent analgesic effects in mice.^23^

The OB in vertebrates carries information to the orbitofrontal cortex, amygdala, and the hippocampus where it is involved in the processing of emotion, fear, and memory. While most memory studies in chronic pain conditions focus on plasticity in limbic structures, we wanted to target the main OB to determine whether chronic pain results in alterations in this particular region that receives a single source of sensory input (olfactory epithelium). Our reported changes in DNA methylation and hydroxymethylation show that the OB itself is modified in chronic pain, thus suggesting an alteration in how odor information is relayed to the limbic system in the first place. To our knowledge, this is the first study documenting epigenetic alterations in a sensory area of the brain that is not directly linked to the pain-causing injury. It is noteworthy that these OB alterations are not linked with how the mice perceive the odors; both injured and control animals prefer or avoid the linalool or 2-methylbutyric acid. However, it is the memory of the unpleasant odor (2-methylbutyric acid) that is augmented in injured mice, suggesting potential alterations both in the OB output through the mitral cells and in the top-down information from the limbic system to the OB. These pain-related changes in the OB are indicative of global sensory alterations that take place once pain becomes chronic; while the initial injury would not directly affect OB plasticity, limbic circuit alterations could in turn modify the epigenetics of other sensory areas, including the OB. This form of “spread” in brain plasticity is one reason why the satisfactory treatment of pain remains elusive.

The increase of DNA methylation within the OB can indicate a series of changes possibly occurring in the injured animals. Given the resolution of our immunoassay, our findings include methylcytosine (mC) in various contexts (CpG and non-CpG) which have been previously described to be prevalent within the OB.^24^ Hypermethylation within OB may be mediated by de novo DNA methylation across whole tissue or convoluted changes in cellular heterogeneity within the tissue^25^ following injury. Similarly, a decrease in hydroxymethylcytosine (HmC) could complement an overall increase of DNA methylation seen in the injured group since HmC prevalence is a substrate for cytosine demethylation.^26^ However, such effects would require more comprehensive analyses within specific cell types and across the whole genome. To date, several studies have demonstrated that DNA methylation dynamics across brain tissues^27^ and within single cells^28^ lend plasticity to neuronal function. Since HmC is enriched in transcriptionally active areas of the genome in the developing mouse and human brain,^29^ we anticipate that a decrease in HmC may lead to transcriptional silencing of genes in injured animals. Our findings hint that similar processes—either through cellular reorganization or through the regulation of DNA methylation machinery—may be an outcome of chronic injury. The data provide an interesting change in the epigenome landscape worthy of further investigation.
